# Risk of hospitalization and death among autistic young people in England during the Covid-19 pandemic

**DOI:** 10.1186/s13229-025-00698-6

**Published:** 2026-01-12

**Authors:** Brigid Saoirse Kennedy, Elizabeth Weir, Matthew C. Fysh, Alex Tsompanidis, Rupert A. Payne, Carrie Allison, Fiona E. Matthews, Simon Baron-Cohen

**Affiliations:** 1 Autism Research Centre, Department of Psychiatry, University of Cambridge, Douglas House, 18b Trumpington Road, Cambridge, CB2 8AH UK; 2 Department of Health and Community Sciences, University of Exeter, Exeter, UK; 3 Institute of Clinical and Applied Health Research, University of Hull, Hull, UK

## Abstract

**Background:**

Autistic people experience increased health vulnerability and risk of premature mortality; the Covid-19 pandemic posed a serious health risk globally. The present study estimated risks of (i) first hospitalization, (ii) first hospitalization with a positive Covid-19 test, (iii) all-cause death, and (iv) Covid-19 associated death from 1 January 2020–31 March 2021 among autistic people compared to matched peers in England.

**Methods:**

We leveraged National Health Service records from 45,756 individuals, including 15,252 autistic individuals, via the Clinical Practice Research Datalink. Participants were matched 1:2 on birth year (± 2 years), gender, and general practitioner practice to 30,504 non-autistic people. The sample primarily comprised males and younger individuals, with a median age of 19.0 years (IQR = 12.0 years), which was expected based on the demographics of clinically diagnosed autistic people. For all outcomes, cox proportional hazards regression models were performed, accounting for matching criteria of gender, birth year, and clustering across GP practices. Additional models adjusting for matching criteria, as well as socioeconomic status, intellectual disability, obesity, alcohol misuse, and smoking were performed to assess risks of all-cause and Covid-19 related hospitalizations. However, due to perfect separation, it was not possible to conduct analyses for mortality that were adjusted for additional covariates (beyond matching factors), and Covid-19 related mortality modelling only assessed risk for male individuals.

**Results:**

Autistic individuals had increased likelihood of all-cause hospitalization (HR: 1.32, 95% CI: 1.22–1.42, *p* < .001) compared to matched peers, even after adjusting for intellectual disability, obesity, alcohol misuse, smoking, socioeconomic status, and matching factors. Autistic individuals had increased risk of Covid-19 related hospitalizations compared to matched non-autistic individuals when only accounting for matching factors; however, after adjusting for additional covariates of interest, autism did not remain a significant predictor of Covid-19 related hospitalization. In addition, while only models adjusting for matching factors could be performed, the results provide some evidence of heightened all-cause mortality risk for autistic individuals compared to matched non-autistic individuals during the covid period (HR = 2.47, 95% CI: 1.31–4.66, *p* = .005) and was inconclusive for Covid-19 associated deaths due to sparse events (HR = 1.26, 95% CI: 0.15–4.14, *p* = .79).

**Limitations:**

The study included a relatively young sample; thus, these results may be less applicable to older individuals and may underestimate effects for older individuals, as increasing age is a well-established risk factor for severe disease due to Covid-19 infection. In addition, due to low counts, analyses for mortality could only be adjusted for matching factors and the Covid-19 mortality analysis could only be conducted among males. Due to the nature of historically collected clinical data, we could not account for all potential confounders (e.g., residence type) and cannot eliminate the possibilities of missingness and/or provider differences in clinical coding.

**Conclusions:**

Autistic young people had increased risks of all-cause hospitalization, with some evidence of increased risks of Covid-19 related hospitalization and all-cause death, during the first 15 months of the Covid-19 pandemic compared to matched non-autistic young people. The study bolsters existing evidence of increased health vulnerability among autistic people, including within the Covid-19 period; however, the results do not provide clarity on whether autism remains an independent predictor of Covid-19 related hospitalization or death. These findings provide the first targeted, clinically-representative, and UK-specific statistics on the health vulnerability of autistic young people during the Covid-19 pandemic, and this issue must be addressed in individual patient care, as well as national and international public health policy.

**Supplementary Information:**

The online version contains supplementary material available at 10.1186/s13229-025-00698-6.

## Background

Autism spectrum conditions (henceforth autism) comprise a set of neurodevelopmental conditions characterized by social communication differences, repetitive behaviors, and restricted interests [1]. Autistic individuals tend to have a cognitive profile with a preference for logical, factual information and are more likely to have differences in sensory perception [1]. Throughout this paper we will use identity first language (e.g., autistic person, autistic people), as this is preferred by the majority of autistic people in the UK, though it should be noted that preferences differ across countries [2, 3]. Autism frequently co-occurs with other neurodevelopmental conditions such as intellectual disability and Attention Deficit/Hyperactivity Disorder (ADHD) [4–6]. The latest prevalence estimates suggest that 1 in 34 children aged 10–14 years are diagnosed as autistic in the UK. Males are more likely to receive an autism diagnosis, with a gender ratio of between 3:1 and 4:1, although this ratio varies depending on age and co-occurring neurodevelopmental diagnoses [6, 7].

Nearly 200,000 people died due to Covid-19 in England from the start of the pandemic to the end of December 2022 [8]. Upward trends or distinct peaks in Covid-19 associated deaths and hospitalizations prompted three distinct, government-ordered lockdowns in England, all of which occurred during the first 15 months of the pandemic (23 March to 23 June 2020, 31 October to 5 December 2020, and 6 January to 26 March, 2021) [9]. Initial public health policy and guidance during this time focused primarily on implementing risk mitigation strategies for individuals known to be at higher risk of severe outcomes from Covid-19 infection, such as older adults and individuals with pre-existing respiratory, cardiovascular, and immunity conditions [10]. In November 2020, UK guidelines were updated to include people with other neurodevelopmental conditions, such as Down Syndrome, in the “extremely vulnerable” category following evidence that such individuals experienced increased risk of hospitalization and death from Covid-19 [11]. 

While autism has traditionally been viewed primarily as a neurocognitive disability, it is now becoming clear that autistic people are more likely to experience a wide range of health vulnerabilities [12–21], potentially putting them at additional risk during public health crises, such as the Covid-19 pandemic. Elevated health risks associated with autism do not only apply to adults, but children and adolescents, too. Both autistic children and adults have higher rates of chronic and sometimes life-threatening physical and mental health conditions involving a range of pathophysiological mechanisms and organ systems [12–14, 16–19, 22–24]. Mental health distress is also common, both during childhood and adulthood, further affecting quality of life [12, 14, 16, 20, 21, 23–25]. For instance, evidence suggests that over 60% of autistic people receive a psychiatric diagnosis before the age of 25 [24], and as many as 1 in 4 autistic adults has attempted suicide [23]. Notably, autistic individuals have higher rates of every condition identified by the UK’s National Health Service for increasing the risk of severe disease from Covid-19 [10], namely respiratory conditions [12, 16, 20, 21], cardiovascular conditions [12–14, 16, 21], diabetes [12–14, 16, 21], kidney disease [12, 17], obesity [12, 14, 16], weakened immune system [12, 17], neurological conditions [12, 14, 17, 19], mental health conditions [12, 14, 20, 21, 23, 24], and substance misuse [21, 24]. Yet, the health vulnerability of autistic people was not specifically addressed in UK public health policies for Covid-19, and there is currently a dearth of evidence surrounding the risk of Covid-19 in autistic people [24].

Recognition of the health vulnerabilities of autistic people exposes major gaps in current provision for clinical care and support for autistic people, generally and in the context of the Covid-19 pandemic. The etiology of these health differences is likely multifactorial in nature, with evidence for biological [26], environmental (adverse life experiences, diet, exercise, sleep) [18, 27–29], and societal factors (unemployment, lower educational attainment, challenges accessing healthcare) [20, 21, 30–33] all likely playing a role. Despite barriers to accessing healthcare [20, 31], autistic people have higher rates of healthcare utilization and healthcare spending, being more likely to have primary care, out-patient, and emergency department visits than others, including increased risk of emergency department visits for psychiatric reasons [21]. Autistic people are also far more likely than non-autistic people to be hospitalized generally [21], for reasons related to cardiovascular disease [12], and for psychiatric reasons [24]. A study of over 400,000 people from Canada suggests that autistic people are 2.75 times more likely to experience all-cause hospitalization [21], and a study of 1.3 million individuals from Sweden suggest that 32 of 100 autistic females and 19 of 100 autistic males, on average, have been hospitalized for a psychiatric reason by the age of 25 [24]. In addition to the conditions listed above that convey increased risk of severe disease from Covid-19, the interplay of these biological, environmental, and structural challenges serve to further escalate risk for autistic children, adolescents, and adults.

Six studies from the United States (US) [34–37], Israel [38], and Korea [39] have identified that autistic people are more likely to experience severe outcomes due to Covid-19, including increased risk of death [34], hospitalization [34–36], ICU admission [37], and mechanical ventilation [38]. One study found that risks of hospitalization and death due to Covid-19 in the US showed a dose-response to the number of co-occurring health conditions, with odds of mortality being 5 times higher for autistic individuals with one co-occurring health condition but 52.5 times higher for autistic individuals with five or more co-occurring health conditions [34].

 The studies above have several key limitations. First, only one of the six studies used medical records across care sectors [38], (as two used hospital data only [36, 37] and three used medical claims data [34, 35, 39]). Second, only two unique datasets have been used across the four US studies (two studies use FAIR Health’s NPIC [34, 35] and two use PINC AI Healthcare data [36, 37]). Third, only one study considered how co-occurring intellectual disability could impact on Covid-19 outcomes [35], finding greater risks among autistic individuals with co-occurring intellectual disability compared to those without [35]. Fourth, one study did not describe the demographic data associated with their sample, thus limiting its contextual relevance [34]. Finally, none of these studies assessed risk of all-cause hospitalization or death during the Covid-19 pandemic, despite guidance from the World Health Organization (WHO) of undercounting of infections/deaths due to a Covid-19 infection [40], poorer healthcare access among autistic people due to the Covid-19 pandemic, and worsening mental health symptoms among autistic people specifically [32, 41, 42]. 

The present study aimed to understand the risks of severe disease from Covid-19 among autistic people. This study is the first to estimate risks of all-cause and Covid-19 specific hospitalizations and deaths among autistic children, adolescents, and adults during the Covid-19 pandemic. Leveraging historical health records from nationally representative primary care data, national hospitalization and death registration data, as well as bespoke data linkages on Covid-19 associated hospitalization, intensive care, and death, this analysis represents the first data from a European population and accounts for a wider range of covariates than existing studies. Furthermore, many Covid-19 studies, and international public health policy during the Covid-19 pandemic, emphasized risks of severe outcomes for older adults in the general population; however, this analysis focuses primarily on estimating risks for autistic children, adolescents, and young adults, as 82.8% of our sample was aged 29 years or younger at the time of the study. In this way, our study provides targeted, nationally representative, and UK-specific estimates of the health vulnerability of autistic young people which can be directly implemented into future national and international public health policies to reduce health-related risks.

## Method

### Study population

The present study included NHS medical records for 45,756 individuals from the Clinical Practice Research Datalink (CPRD), which is a clinically representative database of deidentified National Health Service general practitioner medical records from over 65 million patients across the UK. Each individual in the database is assigned a unique patient identifier which can be cross-referenced across clinical records, death records, hospital records, and Covid-19 specific datasets in England.

Of the 45,756 individuals in our sample, 15,252 had received a clinical diagnosis of autism; the remaining 30,504 patients did not have evidence of autism diagnosis or referral for autism assessment at any time in their health record. The non-autistic participants were matched to the autistic sample 2:1 on birth year (± 2 years), gender, and general practitioner (GP) practice. Because CPRD does not record sex and gender separately (as is the case in many medical record databases and our linked data), these data are based on clinician-report and may refer to either the sex assigned at birth or current gender identity of the individual.

For the purposes of this study, autistic individuals were defined as individuals (1) with at least one relevant autism code (see Appendix A) in their clinical GP record after 1 January 1990, (2) who had clinical follow-up from 1 January 2020, (3) had at least 12 months up-to-standard registration in CPRD, (4) who were in England and eligible for linkage to the Hospital Episode Statistics Admitted Patient Care (HES) dataset [43], Office of National Statistics (ONS) death records [44], Patient-Level 2015 Indices of Multiple Deprivation (IMD) records [45], and Covid-19 specific datasets, (5) who had complete data for the matching criteria and covariates of interest, and (6) had at least two non-autistic matches who were alive on 1 January 2020. 15,252 autistic individuals met these initial criteria. Non-autistic individuals were eligible to be matched if they met criteria 2–5 above and did not have any autism code in their GP record nor siblings or a mother with an autism code in their GP record.

It should be noted that the present study’s sample was taken from a larger CPRD study focusing on longitudinal, health outcomes of autistic people, which did not employ any other exclusion criteria. The larger sample included 120,530 non-autistic people matched 5:1 on age ± 2 years, gender, and GP practice to 24,106 people who were autistic or referred for autism assessment. As the present study specifically focused on autistic individuals and the Covid-19 pandemic period, only individuals with a confirmed autism diagnosis in their clinical record and follow-up from January 1st, 2020, were eligible for inclusion.

**Fig. 1:**
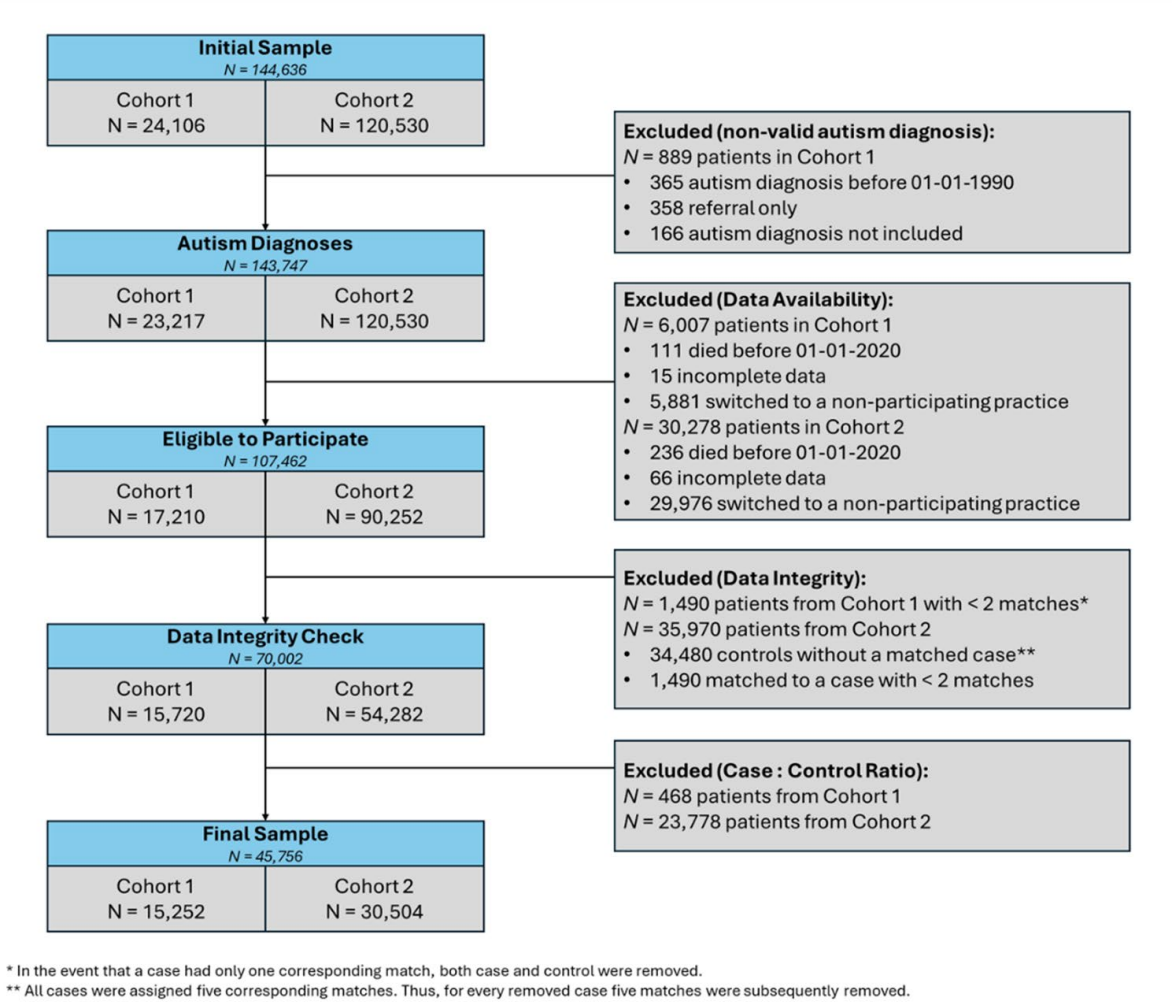
Study flow diagram

Autistic individuals without an autism diagnostic code in their clinical record from January 1st, 1990 were excluded from the present study (*n* = 889 excluded). In addition, as many non-autistic individuals lacked follow-up from January 1st 2020, we determined that the vast majority of our 5:1 matched sets would be excluded. As such, to preserve our sample size, a 2:1 case to control matching system replaced the original 5:1 system, meaning that matched sets could be retained if there was follow-up from January 1st, 2020, for the autistic individual and at least two of their non-autistic matches. If more than two (of the possible five) non-autistic matches had follow-up from January 1st, 2020, the two matches used in the study were randomly selected among the pool. If the autistic individual lacked follow-up from January 1st, 2020, the full matched set (1 autistic individual and all 5 non-autistic individuals per set) was excluded. Full details on exclusion and participant flow are shown in Fig. 1.

### Outcomes and covariates

The four outcomes investigated were all-cause hospitalizations, Covid-19 associated hospitalizations, all-cause death, and Covid-19 associated death. The exposure window was from 1 January 2020 to 31 March 2021 (i.e., 455 days). All-cause hospitalizations and deaths were defined by the date of first hospitalization for any reason or date of death in the participants’ health record for any cause during the study period, including Covid-19 hospitalizations and deaths. Covid-19 hospitalizations and deaths were defined by the date of first hospitalization or date of death in the health record that occurred within 28 days of a positive Covid-19 test during the study period, which is consistent with the National Health Service definition of Covid-19 deaths; [9] a positive Covid-19 test could occur within the hospitalization window (i.e. after the date of admission).

Covid-19 hospitalization data were obtained from three sources: (1) *Covid-19 Hospitalization in England Surveillance System* (CHESS), (2) the *Intensive Care National Audit and Research Centre* (ICNARC), and (3) the HES in conjunction with the *Second Generation Surveillance System* (SGSS). This final source required cross-referencing patient IDs logged alongside positive Covid-19 tests from SGSS to hospital admission recorded in HES. Covid-19 deaths were obtained from sources 1 and 2 above, when Covid-19 was listed as a cause of death in ONS death records, as well as by linking ONS deaths records to positive Covid-19 tests from SGSS, again via the cross-referencing of patient IDs. All individuals who had not died, due to any cause or Covid-19, were censored at the end of the study: 31 March 2021.

Data were also collected regarding obesity, alcohol misuse, smoking, and intellectual disability, using medical code lists that were developed via the CPRD Gold code browser [46]. These code lists were cross-referenced with pre-validated code lists for obesity [47], alcohol misuse [48], smoking [49], and intellectual disability [50], and were subsequently used to leverage data from clinical, test, and referral records to identify individuals with a relevant clinical event (see Appendices B-E). As a crude proxy measure for socioeconomic status, data were leveraged from the 2015 Patient-level English Index of Multiple Deprivation (IMD) which assigns percentile ranks for each individual’s home postcode in England. These percentile ranks were stratified into 4 levels, with lower and higher IMD ranks corresponding to lower and higher SES, respectively.

### Statistical analysis

Group differences in demographics were assessed using t-tests and Chi-Square tests. Adjusted cox proportional hazards regression models were employed to compare outcomes for non-autistic and autistic individuals within the study period, with a significance threshold of α = 0.05 and these were two-sided. For each of the outcomes, Model 1 adjusted for the matching variables of gender and year of birth, as well as the clustering of participants across GP practices. For all-cause hospitalizations and Covid-19 related hospitalizations, an additional Model 2 was included that adjusted for the matching variables, as well as our covariates of interest: smoking, alcohol misuse, intellectual disability, obesity, and socioeconomic status. Due to low counts, it was not possible to run Model 2 for analyses on mortality. In all models, standard errors were clustered by GP practice to account for patient groupings (using the *cluster* function within the cox regression model). All analyses were conducted in RStudio version 4.3.2, using ‘*coxph’ (survival)* and ‘*Surv’ (survminer)* functions.

All models were assessed for violation of the proportional hazard assumption via inspection of Schoenfeld Residuals, and cases in which this assumption was violated are flagged below for transparency. The proportional hazards assumption was violated by the variables of intellectual disability and alcohol misuse in Model 2 of the all-cause hospitalization analysis. A new model that stratified for each of these two variables was conducted and it passed the proportional hazards assumption. As the results did not change, the original model was reported below. Full information on the proportional hazards assumption is provided in Appendix F.

In addition, as all outcomes occurred among male participants, the analysis for Covid-19 related mortality was conducted among males only. The autism variable for this model also violated the proportional hazards assumption that was unable to be resolved. Full information on the proportional hazards assumption is provided in Appendix F.

For the analyses of all-cause and Covid-19 deaths, we attempted to fit Cox models using Firth’s penalised partial likelihood (via the *coxphf* package) to reduce small sample bias. Although the model converged for the coefficient estimates, reliable confidence intervals and p-values could not be computed even after increasing the maximum number of iterations (2,000) and limiting the step size (0.1). This is consistent with the presence of near-monotone likelihood caused by sparse event counts.

Finally, to check the validity of our matching strategy, we conducted a sensitivity analysis in which each analysis was repeated 10 times, each time on a re-sampled set of eligible matched controls using a different random seed. Full results have been reported in Appendix G. In addition, we conducted another sensitivity analysis that included interactions between autism and socioeconomic status, as well as autism and intellectual disability.

## Results

The study sample comprised 45,756 individuals, including 15,252 autistic individuals. The sample was disproportionately male (with a sex ratio of 3.7:1) and 83% of the sample were aged 29 years or younger, with a mean and median sample age of 21.5 years (SD = 12.1) and 19.0 years (IQR = 12.0), respectively. In addition, 22.2% of the autistic group in the sample had co-occurring intellectual disability, which lies between the estimates provided in recent autism prevalence data [6, 7]. In addition, GP practices from the East Midlands were relatively over-represented and GP practices from Greater London, the South West, and the West Midlands were relatively underrepresented among the study sample. See Table 1 for full demographic details of the sample.

**Table 1:** Demographics by diagnostic group

	Autistic People	Non-Autistic People	
	*N* = 15,*252*	*N* = 30,*504*	
**Gender**			
Males (N, %)	11,985 (78.6%)	23,970 (78.6%)	N/A matched
**Age at 1 January 2020**			
Mean (SD)	21.74 (12.06)	21.76 (12.05)	N/A matched
Median (IQR)	19.0 (12.0)	19.0 (12.0)	N/A matched
**Age Categories (years)**			
0–9 (N, %)	1380 (9%)	2739 (9%)	
10–19 (N, %)	6321 (41.4%)	12,636 (41.4%)	
20–29 (N, %)	4954 (32.5%)	9934 (32.6%)	
30–39 (N, %)	1362 (8.9%)	2724 (8.9%)	
40–49 (N, %)	506 (3.3%)	1013 (3.3%)	
50–59 (N, %)	451 (3%)	902 (3%)	
60–69 (N, %)	208 (1.4%)	416 (1.4%)	
70–79 (N, %)	54 (0.4%)	108 (0.4%)	
80+ (N, %)	16 (0.1%)	32 (0.1%)	
**GP Practice Regions**			N/A matched
Region 1 Greater London (N, %)	219 (1.4%)	438 (1.4%)	
Region 2 South East (N, %)	2197 (14.4%)	4394 (14.4%)	
Region 3 South West (N, %)	393 (2.6%)	786 (2.6%)	
Region 4 West Midlands (N, %)	343 (2.2%)	686 (2.2%)	
Region 5 North West (N, %)	2036 (13.3%)	4072 (13.3%)	
Region 6 North East (N, %)	1289 (8.5%)	2578 (8.5%)	
Region 7 Yorkshire and Humber (N, %)	2083 (13.7%)	4166 (13.7%)	
Region 8 East Midlands (N, %)	5049 (33.1%)	10,098 (33.1%)	
Region 9 East of England (N, %)	1643 (10.8%)	3286 (10.8%)	
**IMD Levels**			< 0.001
IMD 1 (most deprived)	3636 (23.8%)	8481 (27.8%)	
IMD 2	3740 (24.5%)	7729 (25.3%)	
IMD 3	3864 (25.3%)	7201 (23.6%)	
IMD 4 (least deprived)	4012 (26.3%)	7093 (23.3%)	
**Intellectual Disability (N**,** %)**	3389 (22.2%)	328 (1.1%)	< 0.001
**Obesity (N**,** %)**	438 (2.9%)	419 (1.4%)	< 0.001
**Alcohol Misuse (N**,** %)**	203 (1.3%)	307 (1%)	0.002
**Smoking (N**,** %)**	1552 (10.2%)	3941 (12.9%)	< 0.001

^a^ General Practitioner

^b^ Indices of Multiple Deprivation

Between 01 January 2020 and 31 March 2021, autistic people were significantly more likely to be admitted to the hospital for any cause than non-autistic people even after adjusting for gender, birth year, SES, obesity, alcohol misuse, smoking, and intellectual disability, and accounting for clustering of participants within GP practice (AHR = 1.32, 95% CI: 1.22–1.42, *p* < .001).^1^ Autistic people were also at greater risk of hospitalization related to a Covid-19 infection than non-autistic people in Model 1 only (HR = 1.74, 95% CI: 1.08–2.82, *p* = .02). However, this effect was attenuated and no longer significant following adjustment for SES, intellectual disability, alcohol misuse, smoking, and obesity (AHR = 1.47, 95% CI: 0.84–2.57, *p* = 0.18).

As noted above, due to a low event count, only a minimally adjusted model was computed for all-cause deaths including birth year and gender, with standard errors clustered by GP practice. These results indicated that autistic people were at greater risk of all-cause death during the Covid-19 period compared to non-autistic people (HR = 2.47, 95% CI: 1.31–4.66, *p* = .005). Comparable effects were produced when a Firth’s penalized Cox model was fitted (HR = 2.46, SE = 0.33, *p* < .001), although confidence intervals could not be reliably estimated due to the sparsity of events^2^.

In addition, those who died from Covid-19 in both the autistic and non-autistic cohorts were exclusively male, thus precluding treatment of gender as a covariate due to perfect separation (in which the models cannot converge due to low counts in at least one variable-level). As such, it was only possible to assess Covid-19 related deaths among males, and the relevant model only adjusted for year of birth with standard errors clustered by GP practice. These results were non-significant (HR = 1.26, 95% CI: 0.15–4.14, *p* = .79) and should be interpreted with caution, as the model violated the proportional hazards assumption. Likewise, a Firth’s penalised likelihood Cox model could not reliably estimate p-values or confidence intervals, but produced a HR with a large standard error, suggesting a highly unstable estimate (HR = 0.91, SE = 0.82)^3^. A full breakdown of results is provided in Table 2, and the modelled effect of all covariates, including matching variables, is provided in Appendix H.

**Table 2:** Frequencies (N) and proportions (%) for autistic and non-autistic people in our sample for the key study outcomes during the study period, as well as minimally adjusted and fully adjusted hazards (HR) for autistic people during the pandemic. Note that all-cause hospitalizations and all-cause deaths include all causes for hospitalization and/or death, including Covid-19

	Non-autistic *N* (%)	Autistic *N* (%)	Model 1^a^		Model 2^b^	
			HR, 95% CI	Sig.	HR, 95% CI	Sig.
**Hospitalizations**						
* All-cause*	1988 (6.52)	1,342 (8.80)	1.37 (1.28, 1.47)	< 0.001	1.32 (1.22, 1.42)	< 0.001
* Covid-19*	38 (0.12)	33 (0.22)	1.74 (1.08, 2.82)	0.024	1.47 (0.84, 2.57)	0.175
**Deaths**						
* All-cause*	17 (< 1)	21 (< 1)	2.47 (1.31, 4.66)	0.005	–	–
* Covid-19* ^*c*^	5 (< 1)	< 5 (< 1)	0.79 (0.15, 4.14)	0.785	–	–

^a^ Minimally adjusted model containing gender and year of birth

^b^ Fully adjusted model containing gender, year of birth, socioeconomic status, intellectual disability, alcohol misuse, obesity, and smoking

^c^ Analysis of Covid-19 deaths contained only year of birth

### Sensitivity analyses

These results were supplemented by a sensitivity analysis for our matching strategy, in which all analyses were repeated 10 times using a randomly resampled set of eligible controls. This produced a near-identical pattern of results, in which autistic individuals were consistently more likely to be hospitalized during the study with Covid-19, as well as for any other reason. In addition, all-cause mortality was significantly more likely in autistic individuals on eight out of 10 runs, and consistently no differences in Covid-19 related mortality were observed. For full information on this sensitivity analysis and its results, see Appendix G.

We also conducted sensitivity analyses exploring the effect of interaction terms between cohort and socioeconomic status, and cohort and intellectual disability for all-cause and Covid-19 hospitalizations. There was no evidence that socioeconomic status interacted with autism for all-cause hospitalizations (AHR = 0.98, 95% CI: 0.92–1.04) or Covid-19 hospitalizations (AHR = 1.04, 95% CI: 0.40–4.51). Conversely, interactions of autism and intellectual disability were present for all-cause hospitalizations (AHR = 0.57, 95% CI: 0.42–0.78); however, individuals with intellectual disability only (AHR = 1.99, 95% CI: 1.47–2.69), autistic people with intellectual disability (AHR = 1.47, 95% CI: 1.31–1.65), and autistic people without intellectual disability (AHR = 1.36, 95% CI: 1.26–1.47) all had higher likelihood of being hospitalized for any cause during the first 15 months of the Covid-19 pandemic than individuals without autism or intellectual disability. Due to perfect separation, estimates including the interaction between autism and intellectual disability could not be computed for Covid-19 related hospitalizations, all-cause deaths, or Covid-19 related deaths during the study period.

## Discussion

Our results highlight the increased health vulnerability among autistic young people compared to non-autistic peers matched on year of birth, gender, and GP practice in the UK. Autistic people had a 32% higher risk of all-cause hospitalization even after adjusting for obesity, intellectual disability, alcohol misuse, smoking, SES, and matching factors. In addition, the study provides some support for increased risks of all-cause death among autistic people compared to matched peers. However, it is not clear whether autism remains an independent risk factor for Covid-19 related hospitalizations among autistic young people, after accounting for key covariates of interest.

Elevated risk of hospitalization related to a Covid-19 infection in autistic people compared to matched peers was observed when adjusting for the matching factors of age, gender, and clustering of individuals across GP practices; however, this effect was attenuated following adjustment for additional covariates, and autism was no longer a significant predictor of Covid-19 related hospitalization in Model 2. This could suggest that autism is not an independent risk factor for Covid-19 related hospitalization. Alternatively, it may be that other sampling-related factors (such as the relatively young age of our participants) precluded us from detecting true effects related to autism, as increasing age is an established risk factor for severe disease due to Covid-19 infection [51]. The existing literature is similarly not conclusive as to whether autism is an independent risk factor regarding co-occurring health conditions, obesity, or intellectual disability. In some studies, autism or neurodevelopmental conditions remain a significant predictor of Covid-19 hospitalization, ICU admission, and mechanical ventilation even after accounting for other co-occurring physical and mental health conditions [36–38]. However, one other study demonstrated a similar pattern to the present analysis after accounting for co-occurring health conditions [34]. Only one previous study accounted for co-occurring intellectual disability in their analyses, and their findings suggested that autistic individuals with and without co-occurring intellectual disability had increased risks of Covid-19 related hospitalization (ORs of 9.3 and 3.6, respectively) [35]. 

Individuals with other types of developmental conditions appear to have increased risks of severe illness and mortality from Covid-19, including people with Down Syndrome [11, 52, 53] and cerebral palsy [54]. Chronic inflammation, immune dysregulation, and respiratory complications are known risk factors for Covid-19 [55] and are also common among autistic people [14, 16], individuals with intellectual disability [16], and people with Down Syndrome [56]. At the same time, systemic factors are also likely to contribute. For instance, a subset of people with developmental conditions may rely on daily visits from support workers and/or live in a residential facility due to their support needs [16], which increases risk of viral exposure. In our sensitivity analysis that included an interaction term for autism and intellectual disability, both autistic individuals with and without co-occurring intellectual disability had increased risks of all-cause hospitalization in the model that included this interaction term. As noted above, this aligns with patterns seen in the only other study that considered the risks of severe disease due to Covid-19 among individuals with autism and co-occurring intellectual disability [35]. 

Future research is needed to understand whether autism is an independent risk factor for severe disease associated with Covid-19 infection, or if elevated risks reported in other papers [34–37, 39] reflect the impacts of previously unaccounted for mediating or confounding factors. This is critical, as these relationships have important implications for public health guidance and clinical care for autistic people. If autism is an independent risk factor for severe disease due to Covid-19 infection, autistic people should be afforded additional supports and designations in the context of future public health crises (e.g., priority vaccination). Conversely, if autism itself is not an independent risk factor and other mediating factors are identified, clinicians should focus their efforts on providing tailored and evidence-based guidance and support to autistic people to reduce their risks for severe outcomes where possible, such as preventing obesity.

Broadly, these findings serve to highlight the health vulnerability of autistic people below the age of 30 years, which has been demonstrated in other studies outside of the context of the Covid-19 pandemic [12, 14, 16–19, 21, 57]. Although the cohorts analyzed in the current study were UK-based, there is good reason to anticipate that our results apply to other countries, too. The balance of evidence from studies conducted in Sweden [15], Canada [21], the USA [28], and the Netherlands [58], for example, reflects that risk of physical health complications is shared by autistic people worldwide. However, the etiologies of observed health vulnerabilities among autistic people are poorly understood, and likely multifactorial in nature. In particular, autistic people may have difficulty engaging with healthcare services due to differences in social communication, sensory sensitivities, difficulty navigating the healthcare system, and anxiety around healthcare appointments [20, 30, 31]. Such findings might partially be explained by a social model of disability, whereby healthcare settings are poorly calibrated for the needs of autistic people by failing to accommodate differences in communication, accessibility, and environment. Yet, at the same time, biological mechanisms have also been associated with both autism and health vulnerability, including immune dysfunction and an increased cardiometabolic burden [12, 14, 17, 18, 21, 27]. These findings underscore the need for improved guidance and training for clinicians on managing health risks for autistic young people.

Considering high rates of mental health conditions and psychiatric hospitalization among autistic people [24], it is critically important that future studies investigate the risks of psychiatric distress and psychiatric hospitalization during Covid-19 as a primary outcome in its own right. Indeed, it has been noted elsewhere that aspects of the lockdown, such as frequent changes to rules, disruptions to routine, uncertainty, and isolation, profoundly impacted the psychological wellbeing of autistic individuals – particularly those with pre-existing mental health conditions [59]. As the present study did not examine this directly, it is possible that results related to all-cause hospitalizations may reflect increased risks related to psychiatric distress among autistic people compared to matched peers.

Clinicians and policymakers should be aware of increased health vulnerability among autistic people and should work collaboratively with autistic individuals to reduce health-related risks. In particular, care must be taken to mitigate risks of severe outcomes for autistic children, adolescents, and young adults. Increased risk of all-cause hospitalization among autistic people is documented in this study, and in several other studies prior to the pandemic, suggesting that the health risks of autistic people are not limited to the Covid-19 period or to Covid-19 specific vulnerabilities [12, 21, 24]. Autistic people and parents of autistic children should be provided with health education on the general health vulnerability of autistic people as part of their post-diagnostic support. Changes to international, national, and local public health policies are needed to improve quality of life of autistic people, who make up 1–3% of the worldwide population.

### Strengths

This retrospective matched cohort study is the first study to date that highlights increased risks of all-cause hospitalization persisted within the pandemic period, even among autistic children, adolescents, and young adults. The present study leverages data from the Clinical Practice Research Datalink [46, 60, 61], a large, nationally representative dataset of medical records in the UK containing historical medical records from 62 million individuals, as well as high quality nationwide linked data on hospitalization (including specialized linkages on Covid-19 associated hospitalization, Covid-19 associated intensive care, and Covid-19 associated death), socioeconomic status, and death. The study includes a large, matched sample and accounts for a wider range of potential covariates than any previous studies (including socioeconomic status, obesity, alcohol misuse, smoking, and intellectual disability)—providing targeted and UK-specific estimates of risk for autistic young people. Its findings suggest that more research is needed to understand whether autism serves as an independent risk factor for severe disease related to Covid-19. In addition, it emphasizes that autistic people remain at increased health vulnerability compared to others and that changes must be made to international, national, and local public health policies to address these risks.

### Limitations

While more comprehensive than existing studies on this topic, there are several key limitations to note:

i) Our study included a relatively young sample of autistic and non-autistic individuals (median age = 20 years). While the study still includes a large number of individuals aged 30 years or older (*n* = 7,708), our results may be less reliable regarding the risks of hospitalization and death during the Covid-19 pandemic among middle-aged and older autistic adults. In particular, as age is a key predictor for severe disease due to Covid-19 [51], death was a relatively rare outcome among members of our sample; thus, only estimates accounting for matching factors were able to be calculated for analyses related to mortality due to perfect separation.
ii) There were violations of the proportional hazards assumption for both intellectual disability and alcohol misuse in Model 2 of our all-cause hospitalization analysis. After stratifying the intellectual disability and alcohol misuse variables, the model passed the proportional hazards assumption. As the results did not change across the original model versus the stratified version, the original results have been reported here. Further information on the proportional hazards assumption has been included in Appendix F.
iii) There were also violations of the proportional hazards assumption for autism in Model 1 of our Covid-19 related death analysis. After further investigation, we determined that all relevant outcomes occurred in male individuals, and we subsetted our analyses to exclude female participants from this analysis only. However, due to low counts, the results from this model were inconclusive and we were unable to resolve the proportional hazards assumption violation. As such, we suggest significant caution in interpreting the findings for Covid-19 related death. Further information on the proportional hazards assumption has been included in Appendix F.
iv) Sex and gender are not recorded separately within the CPRD or in the linked datasets (as is the case with many medical record databases). As gender diversity is over-represented in autism [62, 63], this may affect the validity of our matching criteria based on gender.
v) As outlined in Fig. 1, this sample was taken from a larger, longitudinal dataset developed to understand risks of cardiometabolic outcomes among autistic people. As a result, both autistic and non-autistic matches were excluded from the present sample due to many individuals in the original study lacking follow-up from January 1st, 2020, which may further limit the generalizability of the study. 81 further individuals were excluded due to missing data regarding socioeconomic status.
vi) While our study aimed to assess the impact of obesity, alcohol misuse, and smoking on our outcomes, it was not possible to account for conditions or events that were not recorded in their medical record [64]. As the CPRD data relies on clinical coding, it must be assumed that individuals who lack a relevant code for a particular condition/event have not experienced that condition/event.
vii) The IMD measure of SES, from 2015, was the most recent version of the IMD data when the dataset was received and our study required the creation of a bespoke dataset. However, it should be noted that IMD has high specificity, as it was measured at the patient-level rather than the practice-level, and patient-level IMD data has been shown to be representative of England’s socioeconomic demographics in CPRD studies [65].
viii) While our data are comprehensive, it was not possible to examine all key confounders, such as type of residence. This may be important for understanding individual risks, as individuals living in care homes in England had particularly high risks of death from Covid-19 in the first wave of the pandemic [33].
ix) In December 2020, vaccines for Covid-19 were first made available to some members of the public in England [66]. However, as vaccination status was not provided within healthcare records, we are unable to assess how this impacted the risks of hospitalization and death among autistic and non-autistic people.

## Conclusions

Autistic people in England had increased risks of all-cause hospitalizations compared to non-autistic people matched on birth year, gender, and GP practice during the first 15 months of the pandemic. However, it is not clear whether autism is an independent predictor of Covid-19 related hospitalizations, as these risks were significantly elevated in minimally adjusted models but no longer significant in fully adjusted models after accounting for obesity, intellectual disability, alcohol misuse, smoking, SES, and matching factors. This is the first European study on the topic, and it accounts for a wider range of covariates than previous studies. Taken together with evidence from other studies, our results emphasize that health vulnerability experienced by autistic individuals is not country-specific, but instead widespread. These results underline the importance of changes to international, national, and local public health policies to focus on reducing health vulnerability among autistic people.

## Supplementary Information

Below is the link to the electronic supplementary material.

Supplementary Material 1

## Data Availability

This study is based in part on data from the Clinical Practice Research Datalink obtained under licence from the UK Medicines and Healthcare products Regulatory Agency. The data is provided by patients and collected by the NHS as part of their care and support. It also includes death registration and Indices of Multiple Deprivation data provided by the ONS. The interpretation and conclusions contained in this study are those of the author/s alone. Copyright © (2026), re-used with the permission of The Health & Social Care Information Centre. All rights reserved. The OPCS Classification of Interventions and Procedures, codes, terms and text is Crown copyright (2016) published by Health and Social Care Information Centre, also known as NHS England and licensed under the Open Government Licence available at (https://www.nationalarchives.gov.uk/doc/open-government-licence/open-government-licence.htm) [https://www.nationalarchives.gov.uk/doc/open-government-licence/open-government-licence.htm] (https://www.nationalarchives.gov.uk/doc/open-government-licence/open-government-licence.htm) As data are provided by the Clinical Practice Research Datalink (CPRD), sharing of data is not possible. Parties interested in utilizing data from the CPRD can submit a Research Data Governance Application as follows: (https://cprd.com/data-access) [https://cprd.com/data-access] (https://cprd.com/data-access).

## Footnotes

1 Assessment of Schoenfeld Residuals indicated violation of the proportional hazards assumption in the fully adjusted model for all-cause hospitalizations 𝝌^2^(10) = 22.11, *p* = .015, due to intellectual disability (𝝌^2^(1) = 7.60, *p* = .006) and alcohol misuse (𝝌^2^(1) = 4.30, *p* = .038). All other variables satisfied model assumptions (all 𝝌^2^ ≤ 2.82, all *p*s ≥ 0.121). Rerunning the model adjusted for stratification of intellectual disability and alcohol misuse produced near-identical results for cohort: AHR = 1.37, 95% CI: 1.28–1.47, *p* < .001.

2 This model included cohort, birthyear, and gender, but not practice ID due to computational limitations.

3 This model included cohort and birthyear only, as all covid-19 related deaths were in males, and practice ID was too computationally intensive to include.
