# Supplementary material for: Risk of hospitalization and death among autistic young people in England during the Covid-19 pandemic
Source: Mol Autism. 2026 Jan 12;17:5. doi: 10.1186/s13229-025-00698-6 (PMC12849145; doi:10.1186/s13229-025-00698-6)
Supplement: Supplementary file 1 — Supplementary Material 1 [file 13229_2025_698_MOESM1_ESM.docx]

# Appendices

## ***Appendix A*** Autism code list.

| **Autism Read Codes** | **Autism Read Terms** |
| --- | --- |
| E140.12 | Autism |
| Eu84z11 | [X]Autistic spectrum disorder |
| E140.13 | Childhood autism |
| Eu84012 | [X]Infantile autism |
| Eu84011 | [X]Autistic disorder |
| E140.00 | Infantile autism |
| Eu84000 | [X]Childhood autism |
| E140z00 | Infantile autism NOS |
| Eu84112 | [X]Mental retardation with autistic features |
| E140000 | Active infantile autism |
| Eu84014 | [X]Kanner's syndrome |
| E140.11 | Kanner's syndrome |
| Eu84313 | [X]Heller's syndrome |
| E141.11 | Heller's syndrome |
| E141.00 | Disintegrative psychosis |

## ***Appendix B*** Obesity code list.

| **Med code** | **Read code** | **Read Term** |
| --- | --- | --- |
| 430 | C380.00 | Obesity |
| 3176 | 66C4.00 | Has seen dietician - obesity |
| 7984 | 22A5.11 | O/E - obese |
| 8854 | C380300 | Morbid obesity |
| 11401 | C38z000 | Simple obesity NOS |
| 11461 | 66C..00 | Obesity monitoring |
| 13278 | 22K5.00 | Body mass index 30+ - obesity |
| 16196 | 1444 | H/O: obesity |
| 108478 | 22KE.00 | Obese class III (BMI equal to or greater than 40.0) |
| 59780 | 222A.00 | O/E - obese |
| 22556 | 22K7.00 | Body mass index 40+ - severely obese |
| 108610 | 22KD.00 | Obese class II (body mass index 35.0 - 39.9) |
| 108694 | 22KC.00 | Obese class I (body mass index 30.0 - 34.9) |
| 55585 | 9OK6.00 | Obesity monitoring 3rd letter |
| 40153 | 66CZ.00 | Obesity monitoring NOS |
| 104129 | C380600 | Adult-onset obesity |
| 104421 | C380700 | Lifelong obesity |
| 106771 | C380800 | Childhood obesity |
| 22695 | C380400 | Central obesity |
| 25968 | C380500 | Generalised obesity |
| 55586 | 9OK5.00 | Obesity monitoring 2nd letter |
| 47439 | 9OKA.00 | Obesity monitoring check done |
| 110196 | 66Ce.00 | Telehealth obesity monitoring |
| 70950 | 9OK7.00 | Obesity monitoring verbal inv. |
| 49409 | 9OK4.00 | Obesity monitoring 1st letter |
| 52036 | 9OK3.00 | Obesity monitoring default |
| 52034 | 9OK1.00 | Attends obesity monitoring |
| 67517 | 9OK8.00 | Obesity monitor phone invite |
| 103574 | C38y011 | Obesity hypoventilation syndrome |
| 66406 | C38..00 | Obesity and other hyperalimentation |
| 38059 | C380200 | Extreme obesity with alveolar hypoventilation |
| 17897 | ZV77800 | [V]Screening for obesity |
| 49250 | C380100 | Drug-induced obesity |
| 102514 | 66CN.00 | Risk health associated overweight and obesity, at high risk |
| 106010 | 66CP.00 | Risk health associ overweight and obesity, at very high risk |
| 102150 | 66CM.00 | Risk health associ overweight and obesity, at increased risk |
| 69757 | Cyu7000 | [X]Other obesity |
| 108147 | 8T11.00 | Referral to multidisciplinary obesity clinic |
| 108355 | 8CV7.00 | Anti-obesity drug therapy commenced |
| 21744 | 9OK..11 | Obesity clinic administration |
| 38632 | 66C6.00 | Treatment of obesity started |
| 38799 | C380000 | Obesity due to excess calories |
| 64712 | 66C5.00 | Treatment of obesity changed |

## ***Appendix*** ***C*** Alcohol misuse code list.

| **Med code** | **Read code** | **Read Term** |
| --- | --- | --- |
| 1399 | E23..12 | Alcohol problem drinking |
| 2081 | E23..11 | Alcoholism |
| 669 | E250000 | Nondependent alcohol abuse, unspecified |
| 2082 | E01y000 | Alcohol withdrawal syndrome |
| 2083 | 8BA8.00 | Alcohol detoxification |
| 2084 | E23..00 | Alcohol dependence syndrome |
| 4506 | J153.00 | Alcoholic gastritis |
| 4743 | J612.00 | Alcoholic cirrhosis of liver |
| 3216 | J611.00 | Acute alcoholic hepatitis |
| 4915 | G555.00 | Alcoholic cardiomyopathy |
| 5611 | Eu10.00 | [X]Mental and behavioural disorders due to use of alcohol |
| 5758 | Eu10212 | [X]Chronic alcoholism |
| 6169 | E23z.00 | Alcohol dependence syndrome NOS |
| 6467 | Eu10511 | [X]Alcoholic hallucinosis |
| 7123 | ZV11300 | [V]Personal history of alcoholism |
| 7602 | J617000 | Chronic alcoholic hepatitis |
| 7746 | E250.00 | Nondependent alcohol abuse |
| 7885 | J613.00 | Alcoholic liver damage unspecified |
| 7943 | J617.00 | Alcoholic hepatitis |
| 8030 | ZV6D600 | [V]Alcohol abuse counselling and surveillance |
| 8430 | 1462 | H/O: alcoholism |
| 9489 | 9NN2.00 | Under care of community alcohol team |
| 9508 | Eu10011 | [X]Acute alcoholic drunkenness |
| 9849 | 8H7p.00 | Referral to community alcohol team |
| 11740 | 9k1..00 | Alcohol misuse - enhanced services administration |
| 12442 | 66e..00 | Alcohol disorder monitoring |
| 12974 | E250200 | Nondependent alcohol abuse, episodic |
| 16237 | E01..00 | Alcoholic psychoses |
| 12982 | 136K.00 | Alcohol intake above recommended sensible limits |
| 17607 | Eu10514 | [X]Alcoholic psychosis NOS |
| 18156 | 13Y8.00 | Alcoholics anonymous |
| 19494 | 136S.00 | Hazardous alcohol use |
| 21624 | E230200 | Episodic acute alcoholic intoxication in alcoholism |
| 21650 | 8H35.00 | Admitted to alcohol detoxification centre |
| 23610 | E250100 | Nondependent alcohol abuse, continuous |
| 24064 | E231100 | Continuous chronic alcoholism |
| 24485 | E231300 | Chronic alcoholism in remission |
| 24984 | J671000 | Alcohol-induced chronic pancreatitis |
| 25110 | E013.00 | Alcohol withdrawal hallucinosis |
| 26106 | E231200 | Episodic chronic alcoholism |
| 26323 | Eu10711 | [X]Alcoholic dementia NOS |
| 28150 | E250z00 | Nondependent alcohol abuse NOS |
| 27342 | E012.11 | Alcoholic dementia NOS |
| 28780 | Eu10211 | [X]Alcohol addiction |
| 30162 | Eu10513 | [X]Alcoholic paranoia |
| 30404 | E015.00 | Alcoholic paranoia |
| 30460 | Z4B1.00 | Alcoholism counselling |
| 30604 | F25B.00 | Alcohol-induced epilepsy |
| 30695 | 136T.00 | Harmful alcohol use |
| 31443 | E231.00 | Chronic alcoholism |
| 31569 | E250300 | Nondependent alcohol abuse in remission |
| 32927 | Eu10800 | [X]Alcohol withdrawal-induced seizure |
| 32964 | 66e0.00 | Alcohol abuse monitoring |
| 33635 | E231z00 | Chronic alcoholism NOS |
| 35330 | 9k11.00 | Alcohol consumption counselling |
| 36296 | E230z00 | Acute alcoholic intoxication in alcoholism NOS |
| 36748 | F11x011 | Alcoholic encephalopathy |
| 37691 | Eu10712 | [X]Chronic alcoholic brain syndrome |
| 37946 | E012000 | Chronic alcoholic brain syndrome |
| 38061 | 1B1c.00 | Alcohol induced hallucinations |
| 41920 | E011z00 | Alcohol amnestic syndrome NOS |
| 43193 | E231000 | Unspecified chronic alcoholism |
| 46677 | Z191100 | Alcohol withdrawal regime |
| 47123 | 9k14.00 | Alcohol counselling by other agencies |
| 47555 | F11x000 | Cerebral degeneration due to alcoholism |
| 56410 | 7P22100 | Delivery of rehabilitation for alcohol addiction |
| 57714 | E230.11 | Alcohol dependence with acute alcoholic intoxication |
| 57939 | E014.00 | Pathological alcohol intoxication |
| 59574 | E230300 | Acute alcoholic intoxication in remission, in alcoholism |
| 63529 | 9k12.00 | Alcohol misuse - enhanced service completed |
| 65932 | Eu10512 | [X]Alcoholic jealousy |
| 67651 | E01z.00 | Alcoholic psychosis NOS |
| 68111 | E01yz00 | Other alcoholic psychosis NOS |
| 94553 | 8HkG.00 | Referral to specialist alcohol treatment service |
| 94670 | 136W.00 | Alcohol misuse |
| 95181 | Z191211 | Alcohol reduction programme |
| 96053 | 9k1A.00 | Brief intervention for excessive alcohol consumptn completed |
| 96054 | 9k1B.00 | Extended intervention for excessive alcohol consumptn complt |
| 96993 | 8HkJ.00 | Referral to alcohol brief intervention service |
| 97261 | 8IAF.00 | Brief intervention for excessive alcohol consumptn declined |
| 97680 | 8IAJ.00 | Declined referral to specialist alcohol treatment service |
| 102247 | 8IAt.00 | Extended interven for excessive alcohol consumption declined |
| 104611 | J670800 | Alcohol-induced acute pancreatitis |
| 109108 | 2126C00 | Alcohol dependence resolved |
| 109668 | 8CdK.00 | Specialist alcohol treatment service signposted |
| 109800 | 9NzA.00 | Hospital attendance related to personal alcohol consumption |
| 110494 | 8BAu.00 | Alcohol harm reduction programme |
| 110624 | 8BAs.00 | Alcohol relapse prevention |
| 115844 | 8BAw.00 | Alcohol twelve step programme |
| 12983 | 136E.00 | Ex-very heavy drinker-(>9u/d) |
| 1618 | 1365 | Heavy drinker - 7-9u/day |
| 19493 | 136D.00 | Ex-heavy drinker - (7-9u/day) |
| 8999 | 136P.00 | Heavy drinker |
| 12984 | 136Q.00 | Very heavy drinker |
| 12977 | 1366 | Very heavy drinker - >9u/day |
| 19401 | 136R.00 | Binge drinker |
| 109701 | 9Nz9.00 | Emergency dept attendanc related to personl alcohl consumptn |

## ***Appendix*** ***D*** Smoking code list.

| **Med code** | **Read code** | **Read Term** |
| --- | --- | --- |
| 58597 | 9OO8.00 | Stop smoking monitor phone inv |
| 7130 | 9OO..12 | Stop smoking monitoring admin. |
| 32083 | 9OO..11 | Stop smoking clinic admin. |
| 101338 | 137m.00 | Failed attempt to stop smoking |
| 60720 | 9OO5.00 | Stop smoking monitor 2nd lettr |
| 104086 | 9OOB000 | Stop smoking invitation first SMS text message |
| 106385 | 9OOB200 | Stop smoking invitation third SMS text message |
| 105572 | 9OOB.00 | Stop smoking invitation short message service text message |
| 66387 | 9OO6.00 | Stop smoking monitor 3rd lettr |
| 106384 | 9OOB100 | Stop smoking invitation second SMS text message |
| 12953 | 9OO1.00 | Attends stop smoking monitor. |
| 31114 | 137b.00 | Ready to stop smoking |
| 21637 | 9OOZ.00 | Stop smoking monitor admin.NOS |
| 40417 | 9OO3.00 | Stop smoking monitor default |
| 103507 | 8CdB.00 | Stop smoking service opportunity signposted |
| 19485 | 9OOA.00 | Stop smoking monitor.chck done |
| 53101 | 9OO7.00 | Stop smoking monitor verb.inv. |
| 1822 | 1376 | Very heavy smoker - 40+cigs/d |
| 1878 | 1374 | Moderate smoker - 10-19 cigs/d |
| 12944 | 1373 | Light smoker - 1-9 cigs/day |
| 10558 | 137R.00 | Current smoker |
| 3568 | 1375 | Heavy smoker - 20-39 cigs/day |
| 12955 | 1379 | Ex-moderate smoker (10-19/day) |
| 97210 | 137j.00 | Ex-cigarette smoker |
| 90 | 137S.00 | Ex smoker |
| 93 | 137P.00 | Cigarette smoker |
| 12947 | 137H.00 | Pipe smoker |
| 12943 | 137J.00 | Cigar smoker |
| 1823 | 137P.11 | Smoker |
| 12965 | 137X.00 | Cigarette consumption |
| 12952 | 137Q.00 | Smoking started |
| 12951 | 137Q.11 | Smoking restarted |
| 12966 | 137V.00 | Smoking reduced |
| 11356 | 9N2k.00 | Seen by smoking cessation advisor |
| 12878 | 137T.00 | Date ceased smoking |
| 12942 | 137..11 | Smoker - amount smoked |
| 12957 | 1378 | Ex-light smoker (1-9/day) |
| 12959 | 137B.00 | Ex-very heavy smoker (40+/day) |
| 30762 | 137d.00 | Not interested in stopping smoking |
| 38112 | 13p5.00 | Smoking cessation programme start date |
| 99838 | 137K000 | Recently stopped smoking |
| 104310 | 9ko..11 | Current smoker annual review |
| 105710 | 8HBP.00 | Smoking cessation 12 week follow-up |

***Appendix E*** Read terms for ID.

| **Med code** | **Read code** | **Read Term** |
| --- | --- | --- |
| 11866 | Z7CBE00 | Intellectual functioning disability |
| 110427 | 13VC900 | Intellectual development disorder of unknown aetiology |
| 110833 | PJ50400 | Trisomy 10 |
| 37591 | PJ50100 | Trisomy 7 |
| 32010 | PJ01.00 | Trisomy 21, mosaicism |
| 72139 | PJ10.00 | Trisomy 13, meiotic nondisjunction |
| 114798 | PJ51300 | Trisomy 4p syndrome |
| 67234 | PJ21.00 | Trisomy 18, mosaicism |
| 93133 | PJ22.00 | Trisomy 18, translocation |
| 106114 | PJ51400 | Trisomy 9p syndrome |
| 43565 | PJ11.00 | Trisomy 13, mosaicism |
| 46133 | PJ12.00 | Trisomy 13, translocation |
| 70198 | PJ50600 | Trisomy 12 |
| 107919 | PJ01.11 | Trisomy 21, mitotic nondisjunction |
| 109223 | PJ50311 | Trisomy 9 Mosaic Syndrome |
| 100024 | PJ50800 | Trisomy 22 |
| 42701 | PJ00.00 | Trisomy 21, meiotic nondisjunction |
| 65509 | PJ50300 | Trisomy 9 |
| 61627 | PJ0z.11 | Trisomy 21 NOS |
| 103873 | PJ20.00 | Trisomy 18, meiotic nondisjunction |
| 19038 | PJ1z.11 | Trisomy 13 NOS |
| 61499 | PJ02.00 | Trisomy 21, translocation |
| 18415 | PJ0..12 | Trisomy 21 |
| 46787 | PJ2z.11 | TRISOMY 18 NOS |
| 69476 | PJ50200 | Trisomy 8 |
| 107119 | PJ52z00 | Trisomy of autosomes NEC NOS |
| 1362 | E3...00 | Mental retardation |
| 1787 | E30..00 | Mild mental retardation, IQ in range 50-70 |
| 37867 | E3z..00 | Mental retardation NOS |
| 60473 | Eu71z00 | [X]Mod mental retardation without mention impairment behav |
| 56577 | E31..00 | Other specified mental retardation |
| 42589 | Eu7z.00 | [X]Unspecified mental retardation |
| 51268 | Eu73.00 | [X]Profound mental retardation |
| 302 | E310.00 | Moderate mental retardation, IQ in range 35-49 |
| 4825 | E311.00 | Severe mental retardation, IQ in range 20-34 |
| 6123 | Eu71.00 | [X]Moderate mental retardation |
| 28740 | Eu70.00 | [X]Mild mental retardation |
| 28962 | Eu7..00 | [X]Mental retardation |
| 32820 | Eu7zz00 | [X]Unsp mental retardation without mention impairment behav |
| 34174 | Eu84112 | [X]Mental retardation with autistic features |
| 36143 | Eu72.00 | [X]Severe mental retardation |
| 39016 | Eu70y00 | [X]Mild mental retardation, other impairments of behaviour |
| 42520 | Eu7yy00 | [X]Other mental retardation, other impairments of behaviour |
| 45133 | E312.00 | Profound mental retardation with IQ less than 20 |
| 50606 | Eu70z00 | [X]Mild mental retardation without mention impairment behav |
| 54179 | E31z.00 | Other specified mental retardation NOS |
| 55560 | Eu72z00 | [X]Sev mental retardation without mention impairment behav |
| 55848 | Eu72y00 | [X]Severe mental retardation, other impairments of behaviour |
| 57199 | E3y..00 | Other specified mental retardation |
| 60062 | Eu73z00 | [X]Prfnd mental retardation without mention impairment behav |
| 63273 | Eu7yz00 | [X]Other mental retardation without mention impairment behav |
| 68132 | 6894 | Mental retardation screen |
| 71196 | Eu7y.00 | [X]Other mental retardation |
| 90276 | Eu73y00 | [X]Profound mental retardation, other impairments of behavr |
| 39412 | Eu70100 | [X]Mld mental retard sig impairment behav req attent/treatmt |
| 42886 | Eu7z000 | [X]Unsp mental retard with statement no or min impairm behav |
| 46504 | Eu70000 | [X]Mld mental retard with statement no or min impairm behav |
| 50751 | Eu72100 | [X]Sev mental retard sig impairment behav req attent/treatmt |
| 50947 | Eu72000 | [X]Sev mental retard with statement no or min impairm behav |
| 52602 | Eu84400 | [X]Overactive disorder assoc mental retard/stereotype movts |
| 54881 | Eu71100 | [X]Mod mental retard sig impairment behav req attent/treatmt |
| 56547 | Eu7y100 | [X]Oth mental retard sig impairment behav req attent/treatmt |
| 60913 | Eu71000 | [X]Mod mental retard with statement no or min impairm behav |
| 66383 | Eu7zy00 | [X]Unspecified mental retardatn, other impairments of behav |
| 70008 | Eu7y000 | [X]Oth mental retard with statement no or min impairm behav |
| 2052 | 13Z4E00 | Learning difficulties |
| 56376 | Z7CD200 | Learning difficulties |
| 27533 | C03z.12 | Cretinism |
| 67513 | C03..11 | Cretinism |
| 1278 | E310.11 | Imbecile |
| 2730 | 13Z3.00 | Low I.Q. |
| 4246 | 6664 | Mental handicap problem |
| 4477 | Eu81z11 | [X]Learning disability NOS |
| 4672 | E2F2.00 | Other specific learning difficulty |
| 106219 | 9mA..00 | Learning disability annual health check invitation |
| 106276 | 9mA2200 | Learning disability annual health check invtation 3rd letter |
| 106274 | 9mA2100 | Learning disability annual health check invtation 2nd letter |
| 106249 | 9mA0.00 | Learning disability annual health check verbal invitation |
| 106247 | 9mA2.00 | Learning disability annual health check letter invitation |
| 106248 | 9mA1.00 | Learning disability annual health check telephone invitation |
| 106272 | 9mA2000 | Learning disability annual health check invtation 1st letter |
| 43445 | 9HB2.00 | Learning disabilities health action plan reviewed |
| 100730 | 9HB6.00 | Learning disabilities annual health assessment declined |
| 100965 | 9HB6.11 | Learning disabilities annual health check declined |
| 43447 | 9HB0.00 | Learning disabilities health action plan declined |
| 43436 | 9HB4.00 | Learning disabilities health action plan completed |
| 41391 | 9HB1.00 | Learning disabilities health action plan offered |
| 32511 | 9HB3.00 | Learning disabilities health assessment |
| 19445 | 9HB..00 | Learning disabilities administration status |
| 32952 | 9HB5.00 | Learning disabilities annual health assessment |
| 32667 | 8Hg2.00 | Discharge from learning disability team |
| 107968 | Eu81800 | [X]Specific learning disability |
| 18815 | 8HHP.00 | Referral to learning disability team |
| 19436 | ZS34.11 | Learning disability |
| 22760 | 918e.00 | On learning disability register |
| 96895 | 69DB.00 | Learning disability health examination |
| 98342 | Eu81400 | [X]Moderate learning disability |
| 98293 | Eu81500 | [X]Severe learning disability |
| 99774 | Eu81600 | [X]Mild learning disability |
| 100648 | Eu81700 | [X]Profound learning disability |
| 110002 | 9Nlh.00 | Seen by learning disability team |
| 110792 | 9Nh4.00 | Under care of community learning disability team |
| 108881 | 94Z9.00 | Preferred place of death: learning disability unit |
| 103187 | 8Ce6.00 | Preferred place of care - learning disability unit |
| 51954 | E30..12 | Feeble-minded |
| 84154 | Eu70.11 | [X]Feeble-mindedness |
| 56143 | E141.00 | Disintegrative psychosis |
| 62222 | Eu84312 | [X]Disintegrative psychosis |
| 41207 | E141100 | Residual disintegrative psychoses |
| 68299 | Eu84300 | [X]Other childhood disintegrative disorder |
| 114820 | E141000 | Active disintegrative psychoses |
| 93406 | 9N0y.00 | Seen in learning disabilities clinic |
| 94684 | 8H4f.00 | Referral to learning disabilities psychiatrist |
| 62644 | C372011 | Lesch - Nyhan syndrome |
| 67927 | C372300 | Lesch-Nyhan syndrome |
| 108623 | C372.11 | Lesch - Nyhan syndrome |
| 102234 | 9HB7.11 | Did not attend learning disabilities annual health check |
| 100729 | 9HB7.00 | Did not attend learning disabilities annual health assessmnt |
| 100980 | 9hL..00 | Exception reporting: learning disability quality indicators |
| 27691 | Eu72.11 | [X]Severe mental subnormality |
| 37911 | Eu7z.12 | [X]Mental subnormality NOS |
| 33949 | Eu70.12 | [X]Mild mental subnormality |
| 65468 | Eu73.11 | [X]Profound mental subnormality |
| 34734 | Eu71.11 | [X]Moderate mental subnormality |
| 51622 | E312.11 | Idiocy |
| 46429 | Eu84313 | [X]Heller's syndrome |
| 31599 | E141.11 | Heller's syndrome |
| 31042 | Eu84200 | [X]Rett's syndrome |
| 100507 | PJ33400 | Jacobsen syndrome |
| 98941 | PJ31.11 | Deletion of short arm of chromosome 5 |
| 66566 | PJ32.00 | Deletion of short arm of chromosome 4 |
| 111041 | 9bA0.00 | Mental handicap (specialty) |
| 38954 | ZL9D500 | Seen by psychiatrist for mental handicap |
| 40894 | ZL5B500 | Referral to psychiatrist for mental handicap |
| 32588 | ZL1B500 | Under care of psychiatrist for mental handicap |
| 97406 | ZLD2f00 | Discharge by psychiatrist for mental handicap |
| 32924 | ZLE9400 | Discharge from mental handicap psychiatry service |
| 101999 | Eu84311 | [X]Dementia infantalis |
| 36871 | PJ33300 | Smith-Magenis syndrome |
| 105514 | PJ33A00 | Kleefstra syndrome |
| 106116 | Z7CD211 | LD - Learning difficulties |
| 106704 | P22yz00 | Other reduction deformity of brain NOS |
| 106856 | PJ33900 | Langer-Giedion syndrome |
| 112843 | 38VB.00 | Clinical Outcomes in Routine Evaluatn Learning Disabilities |

***Appendix F*** Assessment of Proportional Hazards Assumption.

|  | **Hospitalizations** | | | | **Deaths** | | | | |
| --- | --- | --- | --- | --- | --- | --- | --- | --- | --- |
|  | **All-cause** | | **Covid-19** | | **All-cause** | | **Covid-19** | | |
|  | ꭓ^2^ | Sig. | ꭓ^2^ | Sig. | ꭓ^2^ | Sig. | ꭓ^2^ | Sig. |  |
| **Model 1** | | | | | | | | |  |
| *Autism* | 2.97 | .085 | 0.33 | .563 | 1.68 | .195 | 4.41 | .036 |  |
| *Birth year* | 0.00 | .967 | 1.23 | .268 | 1.47 | .225 | 1.32 | .251 |  |
| *Gender* | 1.59 | .208 | 0.85 | .356 | 0.41 | .523 | --- | --- |  |
| *Global* | 4.58 | .205 | 2.60 | .457 | 3.52 | .318 | 5.75 | .056 |  |
| **Model 2** | | | | | | | | |  |
| *Autism* | 3.01 | .083 | 0.33 | .564 | --- | --- | --- | --- |  |
| *Birth year* | 0.00 | .952 | 1.30 | .255 | --- | --- | --- | --- |  |
| *Gender* | 1.49 | .222 | 0.85 | .356 | --- | --- | --- | --- |  |
| *SES* | 2.70 | .441 | 0.21 | .976 | --- | --- | --- | --- |  |
| *Smoking* | 0.66 | .417 | 1.04 | .308 | --- | --- | --- | --- |  |
| *Obesity* | 1.74 | .187 | 0.97 | .326 | --- | --- | --- | --- |  |
| *ID* | 7.60 | .006 | 0.35 | .552 | --- | --- | --- | --- |  |
| *Alcohol misuse* | 4.30 | .038 | 0.64 | .425 | --- | --- | --- | --- |  |
| *Global* | 22.11 | .015 | 7.79 | .649 | --- | --- | --- | --- |  |

***Appendix G*** *Sensitivity Analysis on Matching*.

The results of our sensitivity analysis were highly similar to those reported in the manuscript for the majority of trials across all outcomes. In our sensitivity analysis, autistic people remained at increased risk of hospitalization in both the unadjusted model (median HR = 1.33 [IQR = 0.03], *p* < .050 in 10/10 seeds) and the adjusted model (median AHR = 1.29 [IQR = 0.03], *p* < .050 in 10/10 seeds). Likewise, autistic people were at increased risk of covid-19 related hospitalizations in all unadjusted models (median HR = 1.74 [IQR = 0.14], *p* < .050 in 10/10 seeds), but not when adjusting for covariates (median AHR = 1.43 [IQR = 0.16], *p* < .050 in 0/10 seeds). For all-cause death analyses, autistic people were at increased risk of death in 8 out of 10 runs (median HR = 2.34 [IQR = 0.49], *p* < .050 in 8/10 seeds), and no increased risk of death from Covid-19 was observed in any seeds (median HR = 1.00 [IQR = 0.50], *p* < .050 in 0/10 seeds). Full results are provided below.

|  | **Model 1** | |  | **Model 2** | |
| --- | --- | --- | --- | --- | --- |
| **Seed** | **HR, 95% CI** | **Sig** |  | **AHR, 95% CI** | **Sig** |
| *All-Cause Hospitalizations* | | | | | |
| 0 | 1.31 (1.23, 1.40) | < .001 |  | 1.27 (1.18, 1.37) | < .001 |
| 1 | 1.34 (1.25, 1.44) | < .001 |  | 1.30 (1.21, 1.41) | < .001 |
| 2 | 1.33 (1.24, 1.42) | < .001 |  | 1.29 (1.19, 1.39) | < .001 |
| 3 | 1.36 (1.27, 1.45) | < .001 |  | 1.31 (1.21, 1.41) | < .001 |
| 4 | 1.31 (1.22, 1.40) | < .001 |  | 1.27 (1.18, 1.37) | < .001 |
| 5 | 1.33 (1.24, 1.43) | < .001 |  | 1.29 (1.19, 1.39) | < .001 |
| 6 | 1.34 (1.25, 1.44) | < .001 |  | 1.30 (1.21, 1.40) | < .001 |
| 7 | 1.28 (1.20, 1.37) | < .001 |  | 1.24 (1.15, 1.34) | < .001 |
| 8 | 1.32 (1.23, 1.41) | < .001 |  | 1.27 (1.18, 1.37) | < .001 |
| 9 | 1.34 (1.25, 1.43) | < .001 |  | 1.29 (1.20, 1.39) | < .001 |
| *Covid-19 Hospitalisations* | | | | | |
| 0 | 1.70 (1.03, 2.79) | .037 |  | 1.32 (0.71, 2.45) | 0.375 |
| 1 | 1.84 (1.14, 2.96) | .012 |  | 1.44 (0.79, 2.61) | 0.235 |
| 2 | 1.74 (1.05, 2.89) | .032 |  | 1.36 (0.73, 2.55) | 0.336 |
| 3 | 1.70 (1.05, 2.74) | .031 |  | 1.45 (0.83, 2.52) | 0.193 |
| 4 | 1.84 (1.12, 3.01) | .016 |  | 1.58 (0.91, 2.75) | 0.104 |
| 5 | 1.89 (1.15, 3.11) | .012 |  | 1.61 (0.92, 2.81) | 0.092 |
| 6 | 1.95 (1.17, 3.24) | .010 |  | 1.61 (0.90, 2.89) | 0.111 |
| 7 | 1.74 (1.05, 2.89) | .031 |  | 1.42 (0.77, 2.61) | 0.257 |
| 8 | 1.70 (1.03, 2.81) | .039 |  | 1.38 (0.75, 2.55) | 0.299 |
| 9 | 1.65 (1.03, 2.66) | .038 |  | 1.41 (0.81, 2.43) | 0.221 |
| *All-Cause Deaths* | | | | | |
| 0 | 2.49 (1.28, 4.84) | .007 |  | --- | --- |
| 1 | 2.35 (1.24, 4.45) | .009 |  | --- | --- |
| 2 | 3.03 (1.49, 6.15) | .002 |  | --- | --- |
| 3 | 2.34 (1.23, 4.43) | .009 |  | --- | --- |
| 4 | 1.67 (0.93, 3.01) | .085 |  | --- | --- |
| 5 | 1.74 (0.96, 3.15) | .067 |  | --- | --- |
| 6 | 2.00 (1.10, 3.64) | .023 |  | --- | --- |
| 7 | 2.48 (1.30, 4.74) | .006 |  | --- | --- |
| 8 | 2.81 (1.40, 5.64) | .004 |  | --- | --- |
| 9 | 2.00 (1.07, 3.75) | .030 |  | --- | --- |
| *Covid-19 Deaths* | | | | | |
| 0 | 1.33 (0.22, 8.01) | .755 |  | --- | --- |
| 1 | 1.33 (0.22, 8.00) | .754 |  | --- | --- |
| 2 | 2.00 (0.28, 14.29) | .491 |  | --- | --- |
| 3 | 0.80 (0.15, 4.14) | .786 |  | --- | --- |
| 4 | 0.66 (0.13, 3.31) | .617 |  | --- | --- |
| 5 | 0.92 (0.13, 6.57) | .938 |  | --- | --- |
| 6 | 1.00 (0.18, 5.48) | .997 |  | --- | --- |
| 7 | 1.00 (0.18, 5.48) | .996 |  | --- | --- |
| 8 | 1.33 (0.22, 8.05) | .759 |  | --- | --- |
| 9 | 0.66 (0.13, 3.31) | .617 |  | --- | --- |

***Appendix H*** *Full model specification*.

| **All Cause Hospitalizations** | | | |
| --- | --- | --- | --- |
| **Model 1 (minimally adjusted)** | | | |
|  | **HR** | **Sig.** | **95% CI** |
| *Autism* | 1.37 | < .001 | (1.28, 1.47) |
| **Model 2 (fully adjusted)** | | | |
|  | **AHR** | **Sig.** | **95% CI** |
| *Autism* | 1.32 | < .001 | (1.22, 1.42) |
| *Smoking* | 1.48 | < .001 | (1.34, 1.63) |
| *Obesity* | 1.45 | < .001 | (1.21, 1.74) |
| *ID* | 1.17 | 0.01 | (1.03, 1.32) |
| *Alcohol Misuse* | 1.47 | 0.00 | (1.15, 1.87) |
| *IMD Quartile 2* | 1.08 | 0.13 | (0.98, 1.19) |
| *IMD Quartile 3* | 1.13 | 0.02 | (1.02, 1.26) |
| *IMD Quartile 4* | 1.28 | < .001 | (1.14, 1.44) |
|  |  |  |  |
| **Covid-19 Hospitalizations** | | | |
| **Model 1 (minimally adjusted)** | | | |
|  | **HR** | **Sig.** | **95% CI** |
| *Autism* | 1.74 | 0.02 | (1.08, 2.82) |
| **Model 2 (fully adjusted)** | | | |
|  | **AHR** | **Sig.** | **95% CI** |
| *Autism* | 1.47 | 0.17 | (0.84, 2.57) |
| *Smoking* | 1.23 | 0.47 | (0.70, 2.15) |
| *Obesity* | 1.88 | 0.17 | (0.77, 4.59) |
| *ID* | 1.46 | 0.32 | (0.70, 3.04) |
| *Alcohol Misuse* | 1.67 | 0.33 | (0.60, 4.67) |
| *IMD Quartile 2* | 0.60 | 0.18 | (0.29, 1.26) |
| *IMD Quartile 3* | 1.32 | 0.35 | (0.73, 2.40) |
| *IMD Quartile 4* | 1.10 | 0.79 | (0.55, 2.21) |
|  |  |  |  |
| **All-Cause Deaths** | | | |
| **Model 1 (minimally adjusted)** | | | |
|  | **HR** | **Sig.** | **95% CI** |
| *Autism* | 2.47 | 0.005 | (1.31, 4.66) |
| **Covid-19 Deaths** | | | |
| **Model 1 (minimally adjusted)** | | | |
|  | **HR** | **Sig.** | **95% CI** |
| *Autism* | 0.79 | 0.79 | (0.15, 4.14) |
